# The effect of industrial and urban dust pollution on the ecophysiology and leaf element concentration of *Tilia cordata* Mill.

**DOI:** 10.1007/s11356-024-34999-9

**Published:** 2024-09-24

**Authors:** Karolina Bierza, Wojciech Bierza

**Affiliations:** Institute of Biology, Biotechnology and Environmental Protection, Faculty of Natural Sciences, University of Silesia in Katowice, Bankowa 9, 40-007 Katowice, Poland

**Keywords:** Chlorophyll content, Functional traits, Heavy metals, Urban ecology, Air-originated metals (AOM), Bioindication

## Abstract

The influences of airborne trace elements in urban dust on element concentrations and functional traits of *Tilia cordata* were examined. For the present study, the unwashed and washed leaves of *T. cordata* were collected to assess the concentration of metals in Katowice City, Poland, from sites of different traffic intensity and industry activity. The content of Al, Cd, Cr, Cu, Fe, Mn, Pb, Zn, C, and N was measured. Additionally, a number of functional traits such as photosynthetic pigment content, specific leaf area (SLA), leaf dry matter content (LDMC), and diseased areas of the leaves were determined to assess the impact of the polluters on the physiology of the trees and their resources acquisition strategy. We hypothesized that the photosynthetic pigments of *T. cordata* will decrease with the traffic and industry intensity, and the traits related to the resources acquisition and stress resistance will shift into a more conservative strategy. The Principal Component Analysis and the Inverse Distance Weighting (IDW) interpolation method helped to identify that the Fe, Zn, Al, and Cr were related mainly to traffic intensification and Pb to industrial activities. The results indicate that Katowice is considerably polluted by Zn (up to 189.6 and 260.2 mg kg^−1^ in washed and unwashed leaves, respectively), Pb (up to 51.7 and 133.6 mg kg^−1^), and Cd (up to 2.27 and 2.43 mg kg^−1^) compared to other cities worldwide. Also, a reduction of approximately 27% in the photosynthetic pigments was observed at the high-traffic and industrial sites. The trees from the mainly affected areas with heavy traffic and industry tend to apply a conservative resources strategy with a decrement in SLA and an increment in LDMC. In contrast, the opposite trend was observed at the less affected sites (high SLA, low LDMC). The study showed that unfavourable urban conditions can trigger a plastic response on multiple levels. Knowledge of the possible paths of adaptation to urban conditions of different plant species is nowadays crucial to appropriate urban greenery planning.

**Supplementary Information:**

The online version contains supplementary material available at 10.1007/s11356-024-34999-9.

## Introduction

The rapid growth of cities, industry, and transport has significantly deteriorated environmental quality, especially in areas with the highest population density. It applies to water, soils, and air, especially in urban areas. Combustion processes associated with the industry, the municipal sector, and transport are the source of a considerable number of pollutants, which are highly heterogeneous in terms of chemical composition. Among them is particulate matter (PM), a mixture of solid and liquid particles with different diameters and chemical properties that are highly hazardous to human health (Zhang et al. 2015; Zhou et al. 2020). The components of PM are typically organic and elemental carbon, nitrates (V), sulphates (VI), Br, Cl, Na, K ions, and many trace elements such as Al and heavy metals (HMs) (Yang et al. 2018). The types of pollutants emitted depend primarily on local human activities and transport intensity (Li et al. 2019). Exhaust and non-exhaust emissions from road traffic and the combustion of solid biomass are the primary sources of PM in European city centres (Segersson et al. 2017; Johansson et al. 2009). These first are generated during fuel combustion; the latter is related to the tire’s wear, brakes, clutch, and road surface (Piscitello et al. 2021). Various components, such as steel, glass fibres, or plastics, are commonly used in vehicle brake linings. However, also significant amounts of Cu, Sb, Ba, Al, Si, Ti, Zn, Ni, Cr, and Pb and a small amount of Cd have been reported in brake dust (Sinha et al. 2020; Adamiec et al. 2016). Tire wear and tread and road surface abrasion are sources of HMs such as Al, Sb, Bi, Cu, Fe, Mn, Ni, Ti, V, and Zn (Panko et al. 2018); thus, HM pollution is usually connected with the higher traffic typical for urban areas (Alatou and Sahli 2019; Sevik et al. 2019; Alexandrino et al. 2020; Xu et al. 2023). These particles emitted into the atmosphere can persist for a long time until they are eventually deposited as dust on streets, buildings, or plants (Zhang et al. 2006). The elevated level of HM pollution found in urban dust can also threaten humans even years after eliminating the source of pollution (Wiechuła et al. 2006).

Because plants are considered to be an effective entrapping surface for dust (Grote et al. 2016; Chaudhary and Rathore 2019), they are often used as indicators of trace element pollution (Santos et al. 2019; Fang et al. 2021; Levei et al. 2021; Esfandiari and Hakimzadeh 2022). However, the anthropogenic particles may affect the plants at the morphological and physiological level, for example, influencing the photosynthesis process by altering the optical properties of the leaves and reducing the level of radiation reaching the chloroplasts by reflecting or absorbing it (Naidoo and Chirkoot 2004; Nanos and Ilias 2007), clogging the stomata, which reduces stomatal conductance (Chaurasia et al. 2022); also the concentration of chlorophylls and carotenoids may be reduced due to various chemical reactions (Javanmard et al. 2020; Dhal et al. 2024). The composition of dust will also affect plant health due to the possible transfer of elements into the leaf tissues—mainly by adsorption and internalization through the cuticle and penetration through the stomata (Shahid et al. 2017; Sharma et al. 2020b). In addition, dust deposited on the soil also enters plants through the roots. Heavy metals present in dust, such as cadmium (Cd), lead (Pb), chromium (Cr), zinc (Zn), and mercury (Hg), disrupt many biochemical and metabolic processes (Sharma et al. 2020a). These include substituting essential elements in biomolecules, damage to cell membranes and changes in transcription patterns, blocking of enzyme functional groups, and generating ROS, which cause oxidative damage and can also lead to disruption of the electron transport mechanism in chloroplast and mitochondrial membranes (André et al. 2006; Pandey and Tripathi 2011; El-Khatib et al. 2020).

Moreover, the increase of necrosis and chlorosis on leaves of trees in polluted areas was found by Khavaninzadeh et al. (2014). HMs were found to affect the leaf epidermis firstly, causing injuries and alterations in leaf structure, thereby decreasing photosynthesis levels and impairing the leaf physiology; in some cases, the injuries also occurred at the epidermis at the bottom layer of the leaf (Rai et al. 2010; Barış Özel et al. 2015). Many studies also report the reduction of chlorophyll and carotenoid contents in plants growing in urban areas (Khosropour et al. 2019; Uka et al. 2021; You et al. 2021). As a result of the multidirectional alterations in leaf structure and physiology induced by pollutants, plants may change their resource management strategy due to their plasticity. Traits such as leaf dry matter content (LDMC), specific leaf area (SLA), and leaf thickness (LT) have been found to be good predictors of resource capture, usage, and availability (Wilson et al. 1999; Wright et al. 2004). Mukherjee and Agrawal (2018) and Zhu and Xu (2021) showed significant shifts in these traits in urban trees. The relationship between the abovementioned parameters and the pollution level of the environment suggests that they can be used as a tool for determining the effect of pollutants on plants and a feature suitable for biomonitoring purposes.

While the studies on HM content in different plant species, also presenting the spatial distribution under urban environments, are numerous, there is a need to gather more information about the effect of these pollutants on the biological response of plants regarding functional traits. In this study, we aimed to determine the pollution level in Katowice, Poland, especially according to traffic and industry intensity, and verify whether selected functional traits of *Tilia cordata* Mill. reflect the pollution level. We expect that the levels of traffic-related elements (Fe, Zn) will be connected with the city centre, while Pb and Cd will dominate in the leaves of trees growing in the post-industrial district. We also hypothesize that the trees from the industrial district and heavy traffic areas will exhibit a decrease in photosynthetic pigment content, as well as higher investment in leaf structure displayed by lower SLA and higher LDMC as a response to harsh conditions (higher temperature in the strict, city centre, large proportion of paved surface, higher pollution).

## Materials and methods

### Study site

The sampling was conducted within the city of Katowice, Poland, the capital of the Silesia province. The industrial revolution in the Katowice region caused rapid development of industry, mainly coal mining, the metal industry, and machine construction. This resulted in the formation of dozens of towns and urbanized municipalities, which in 1960 and 1970 were finally integrated into one industrial and mining city with an area of 164.64 km^2^ (Krzysztofik et al. 2019). In 2017, the number of Katowice inhabitants was 1010 per km^2^, and the number of registered vehicles reached the number of over 260,000, which is 1584 vehicles per km^2^. The total road network length of 83.15 km per km^2^ of the city area was the highest among the Polish cities (Caban 2021). The study was conducted in the city centre and a former industrial district, Katowice-Szopienice, where a non-ferrous metal smelter existed from 1834 to 2008. For over 100 years, the smelter has been producing Zn and Cd; since the 1940s, the Cu refinery was constructed, and additionally, in 1955, Ag production started (Zieleziński 2023). The city is located in a temperate transitional climate zone, with a predominantly influence of oceanic masses, moderating the climate in winter and cooling it in summer. The mean annual temperature is 7.9 °C, the total annual precipitation is 724 mm, and the dominant wind directions are southwest and northwest (City of Katowice 2012).

### Plant species

*Tilia cordata* Mill. (Malvaceae) was used to evaluate the levels of metals in areas differing with traffic and industrialization intensity. Small-leaved linden is a popular tree planted in Western, Nordic, and Central European cities and parks (Stravinskienė et al. 2015). This deciduous species is a large tree of variable form with a hemispherical crown up to 20 m high. The leaf lamina is suborbicular, 3–8 cm in diameter, abruptly acuminate, cordate at the base, and the margin is dentate except the basal sinus. Leaves have no simple hairs at maturity but patches of reddish-brown tufted hairs on the basal parts of the main veins. *T. cordata* is one of the most widely distributed trees of the temperate lowlands of Europe and a small part of western Siberia. Small-leaved linden is sensitive to drought but tolerant to very low temperatures and is regarded as tolerant of urban pollution (Pigott 1991).

### Sampling

**Fig. 1:**
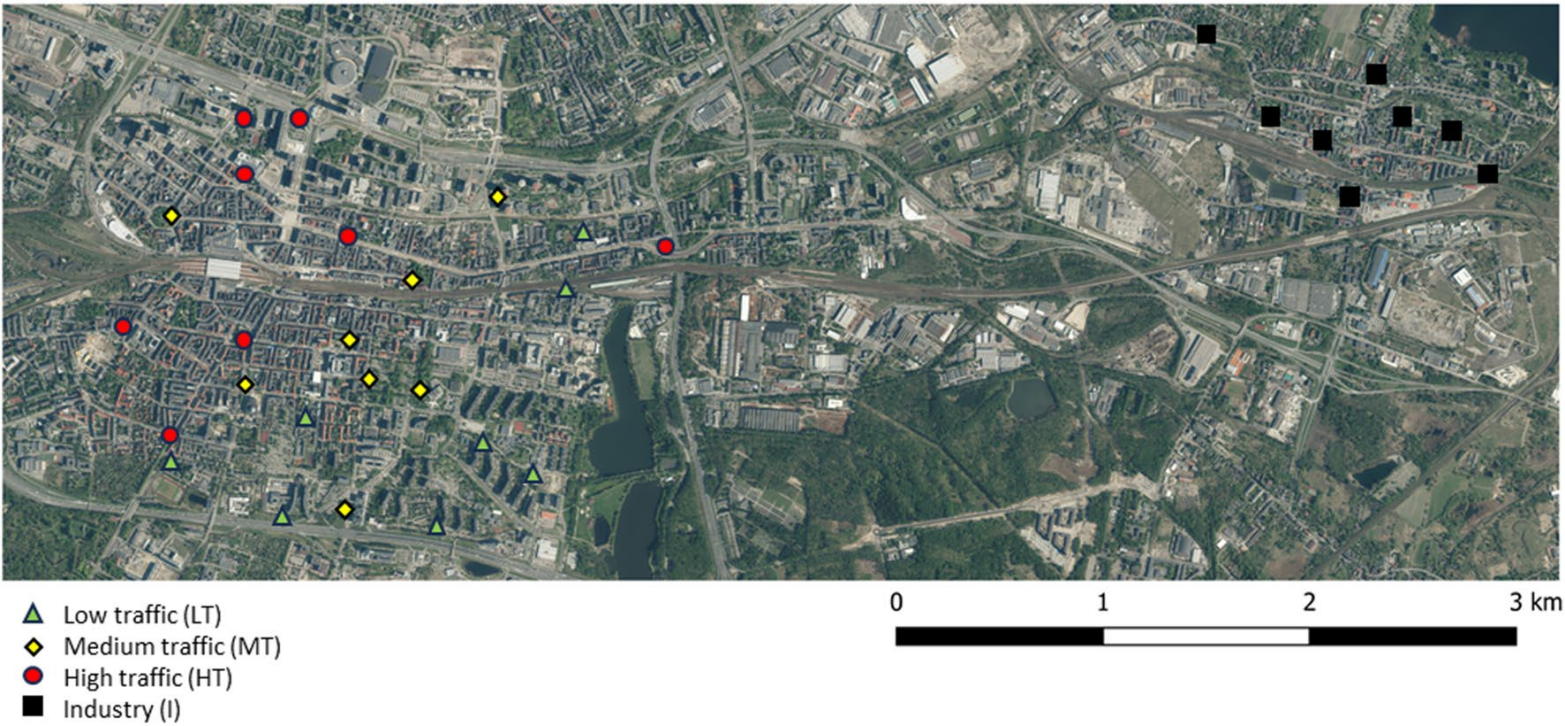
Sampling points within Katowice city

The sampling occurred in August 2017, at least 5 days without rain before sampling. The sampling was conducted at 24 points in the Katowice centre and eight points in the post-industrial district of Katowice-Szopienice, where *T. cordata* trees were present and at comparable size and age, and the outer part of the crown was accessible. The sampling sites in the city centre were divided according to traffic intensity into three groups (eight sampling points per group): low traffic (LT)—which are main roads in urban settlements; medium traffic (MT)—streets in the city centre; and high traffic (HT)—major streets with heavy traffic including bus stops and tramway lines. The Katowice-Szopienice district’s sampling sites were considered heavily polluted primarily by industry (I) (Fig. 1). The leaves were collected from all directions from the trees. Fully developed leaves (*n* = 25) were collected at 2-m height from the outer part of the crown. Leaves were placed in sealed plastic bags and transported to the laboratory in cooling bags to avoid dehydration. In the laboratory, 20 leaves were subjected to morphological measurements, and 5 leaves were selected for photosynthetic pigment measurements. Additionally approx. 250 g of leaves were collected for the chemical analyses.

### Determination of leaf functional traits

Before the analyses, the leaves were washed under deionized water and dried with paper towels. Leaf relative water content (RWC, %) was calculated as follows (Eq. 1) for 20 randomly chosen leaves per sampling point:

1 $$RWC = (FW -DW /TW- DW) \times 100$$

where FM is leaf fresh mass, DW is leaf dry mass, and TW is leaf turgor mass–water-saturated leaf weight measured after 10–12 h in water saturating conditions (petiole in water). The leaves were scanned, and the leaf area (LA) was measured using ImageJ software. Then, the leaves were dried in the oven at 80 °C until the stable dry weight was reached. Specific leaf area (SLA), leaf dry matter content (LDMC), and leaf thickness (LT) were measured on the same leaves collected for RWC according to Eqs. (2, 3, 4) (Perez-Harguindeguy et al. 2013).

2 $$SLA = LA/DW [{\text{mm}}^{2} {\text{mg}}^{-1}]$$

3 $$LDMC = DW/FW[\text{mg }{\text{g}}^{-1}]$$

4 $$LT =\frac{ 1}{SLAxLDMC}$$

### Quantifying leaf disease damage

**Fig. 2:**
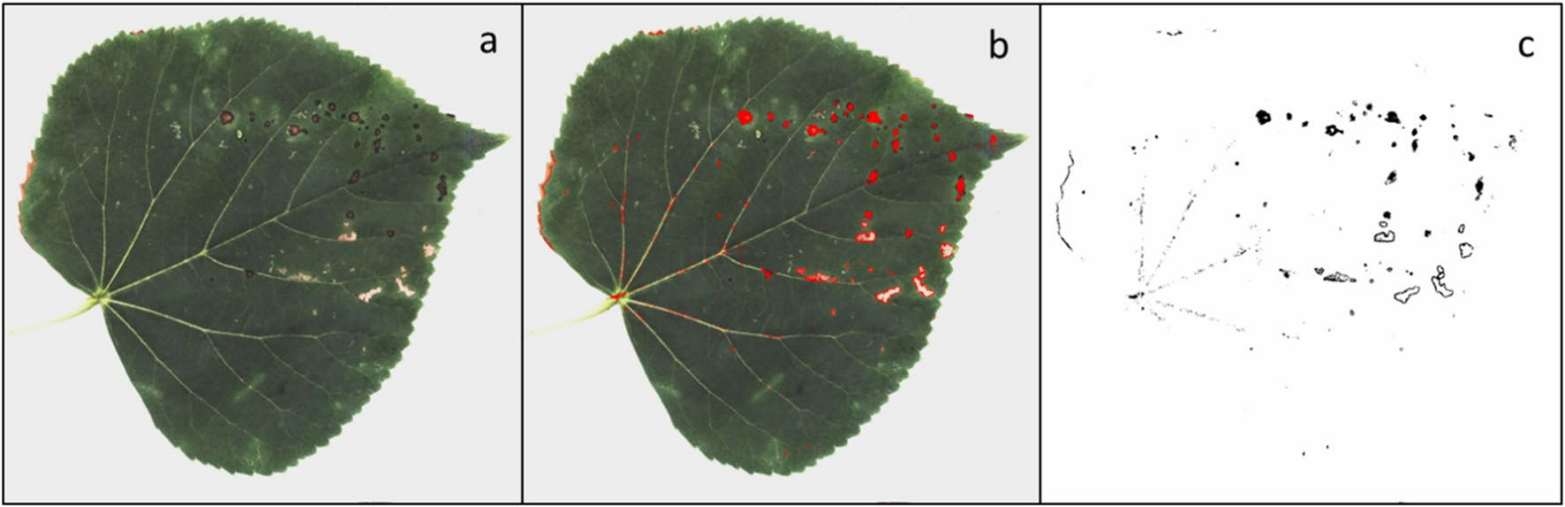
Three steps to quantify the damaged area of the leaves (**a** scan of the leaf, **b** marking the damaged sections, **c** analyzing the damaged area)

The damaged area of leaves was measured using image analysis in ImageJ (public domain software), according to Pride et al. (2020). The leaves were scanned in a resolution of 600 dots per inch (dpi); subsequently, the images were imported to the ImageJ software. The next step was the measurements of the whole leaf area, followed by the measurements of the diseased leaf area by adjusting colour threshold parameters for the hue range from 0 to 50, which optimally covered the diseased area. The percentage of the damaged leaf was then calculated (Fig. 2).

### Pigments content measurements

The total chlorophyll (Chl_tot_) content, as well as chlorophyll *a* and *b* (Chl_a_, Chl_b_) and carotenoids (Car), were determined according to Pompelli et al. (2013) and Richardson et al. (2002). The 100 mg fresh washed leaf sample was extracted in 5 ml of preheated DMSO (dimethyl sulfoxide) to 65 °C for 24, 48, 72, and 96 h to examine which time is optimal for the highest extraction efficiency for *Tilia cordata* tissues. The 72 h were optimal to obtain the best extraction efficiency. Three milliliters of the solvent was then transferred to glass cuvettes. The spectrophotometer was calibrated to zero absorbance using a blank of pure DMSO. The content of chlorophylls and carotenoids in samples and in blank was measured using a spectrophotometer (Hach Lange D5000) at *A*_665_, *A*_649_, *A*_480_, *A*_663_, and *A*_645_ and calculated according to Eqs. (5–8) (Pompelli et al. 2013; Richardson et al. 2002). All measurements were done in 3 replicates.

5 $${Chl}_{a} = (12.19 {A}_{665})-(3.45 {A}_{649})$$

6 $${Chl}_{b} = (\text{21,99} {A}_{649}) - (\text{5,32} {A}_{665})$$

7 $${Chl}_{tot} = (0.0202 {A}_{645}) + (0.00802{ A}_{663})$$

8 $$Car = \frac{(1000 {A}_{480}) - (2.14 {Chl}_{a}) - (70.16{ Chl}_{b})}{220}$$

### Element content

Part of the collected leaves (approx. 100 g) was washed first with tap water, then under deionized water, and the rest was left unwashed. The leaves were then oven-dried at 70 °C for 72 h and mortar ground to fine powder. The material was mineralized in a mixture of concentrated nitric acid and 30% hydroxyl peroxide (4:1 v/v) in a microwave oven (Ethos One, Milestone, Italy). Mineralization was carried out at 190 °C with a heating ramp of 10 min and a holding time of 30 min. The obtained solution was filtered, and the final volume was adjusted to 25 ml with distilled water. The filtrates were analyzed for the presence of Al, Cd, Cr, Cu, Fe, Mn, Pb, and Zn using flame absorption spectrometry (iCE 3500, Thermo Fisher Scientific Inc., USA). To ensure the quality of the analyses, all of the analysis procedures included blank samples (distilled water) and certified reference material (Oriental Basma Tobacco Leaves [INCT-OBTL-5], Institute of Nuclear Chemistry and Technology, Poland). The levels (%) of N and C in dried plant material were measured with a CNS analyzer (Variomax CNS, Elementar Analysensysteme, GmbH, Germany).

### Air-originated metals (AOM)

AOM has been used to evaluate the contribution ratio of the metals originating from the atmospheric sources (Safari et al. 2018; Hatami-manesh et al. 2021). The AOM factor was estimated using Eq. (9).

9 $$AOM=\left(\frac{\left[C\right]unwashed-\left[C\right]washed}{\left[C\right]unwashed}\right)\times 100[\%]$$

where [*C*]_washed_ and [*C*]_unwashed_ are the element contents in the washed and unwashed leaf samples, respectively.

### Statistical analysis

The Principal Component Analysis (PCA) has been used to determine the relationship between the investigated elements, which can identify their origin sources (Yongming et al. 2006; Zgłobicki et al. 2019). The differences between the concentrations of elements in plant tissues were characterized by one-way ANOVA and post-hoc LSD Fisher test prior to analysis of the normality of data distribution (Kolmogorov–Smirnov test) and homogeneity of variance between the distinguished groups (Levene test). The differences between concentrations in washed and unwashed leaves were tested by *t*-Student’s test (*p* < 0.05). The statistical analyses were performed using CANOCO ver. 4.5 (ter Braak and Šmilauer 2002) and Statistica v. 13.0 (Dell Inc.).

### Geographic information system

The data obtained on the element concentrations in the AOM and selected functional traits of *T. cordata* at the study area were used to generate distribution patterns reflecting the particulate pollution in Katowice and its influence on the morpho-physiology of linden. The Inverse Distance Weighting (IDW) function implemented in Qgis (version 3.22) was used to perform the analysis. It enabled to produce a heat map of the metal concentrations by spatial interpolation. The IDW method calculates a value for each point based on the values of adjacent points weighted by the inverse of their distance. As a result, the more distant a point is, the less influence it has on the interpolated value, according to Eq. 10:

10 $$Zp=\frac{{\sum }_{t=1}^{n}(\frac{{z}^{i}}{{d}_{i}^{p}})}{{\sum }_{t=1}^{n}(\frac{1}{{d}_{i}^{p}})}$$

where *Z*_*p*_ is the interpolated value of the concentrations/ traits, *Z*_*i*_ is the actual measured values, *n* is the number of sites considered (here *n* = 24 for Katowice centre and* n* = 8 for Katowice-Szopienice), and *d* is the distance between the locations, while the *p* value defines the smoothness of the interpolation and commonly the used value is 2, as also in this case (Alexandrino et al. 2020; Xie et al. 2017).

## Results and discussion

### Trace element concentrations in *T. cordata* leaves

**Fig. 3:**
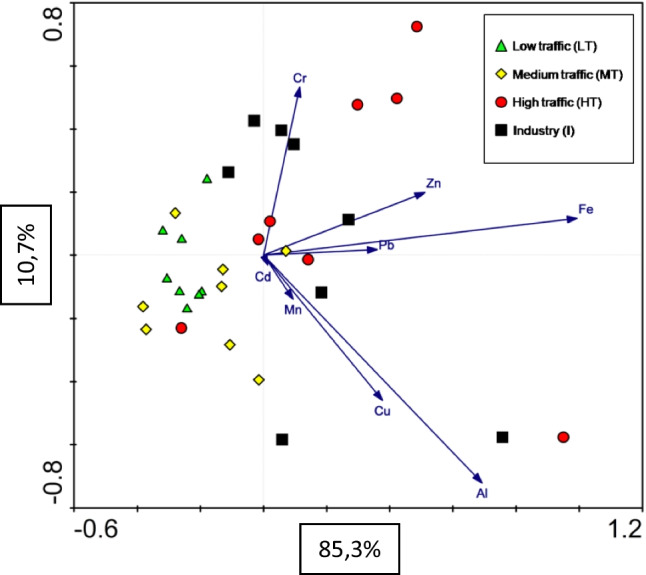
Principal Component Analysis (PCA) for metal concentrations found in unwashed *Tilia cordata* leaves at sampling points from different pollution categories (LT, low traffic; MT, medium traffic; HT, high traffic; I, post-industrial)

The results presented in Table 1 show the elements’ mean concentrations and standard errors in the unwashed and washed leaf samples of *T. cordata* for different vehicular traffic intensities and post-industrial zones. The statistically significant differences between the concentrations in unwashed and washed linden leaves concerned Fe, Al, Pb, Zn, Cr, and Cu in at least two land categories and suggest their air origin. Fe and Zn dominated at sites connected to high traffic and industry, and Pb at industry sites. In order to further investigate the origin of the elements, the biplot, based on the first two principal components of the PCA analysis conducted on the concentrations of metals in unwashed leaves, is presented in Fig. 3. The analysis explains 96% of the total variability. The LT and MT classes can be characterized by the lowest concentrations of Al, Fe, and Zn. In contrast, the highest concentrations of these elements are present in samples from HT sampling points. Also, Cr is associated with the HT and I points. The first axis is connected to the Fe, Zn, and Pb gradients, while the second is related to the Cr and Cu gradients.

**Table 1:** Mean ± SE values [mg kg^-1^] of trace elements in unwashed and washed *T. cordata* leaves

Pollution class	Fe		Al		Mn		Zn		Pb		Cr		Cu		Cd	
	Mean	SE	Mean	SE	Mean	SE	Mean	SE	Mean	SE	Mean	SE	Mean	SE	Mean	SE
Unwashed																
LT	347.48 Ab	16.54	83.55 Aa	15.00	68.88 Aa	19.50	60.00 Aa	5.89	25.19 Aa	0.90	12.37 Aa	1.30	10.51 Aa	0.87	1.51 Aab	0.15
MT	402.26 Ab	48.44	136.47 Aa	24.93	71.36 Aa	13.15	64.90 Aa	5.81	24.64 Aa	1.31	11.06 Aa	1.95	11.50 Aa	0.81	1.43 Aab	0.08
HT	778.43 Aa	111.16	180.40 Aa	59.15	61.10 Aa	6.87	122.44 Ab	22.00	29.70 Aa	2.44	12.85 Aa	1.53	11.08 Aa	0.84	2.54 Aa	1.35
I	687.73 Aa	70.54	180.97 Aa	63.96	73.99 Aa	11.28	116.38 Ab	14.49	76.19 Ab	12.97	15.09 Aa	1.58	10.27 Ab	0.95	1.71 Ab	0.17
Washed																
LT	116.65 Bb	4.80	45.39 Aa	13.67	52.09 Aa	12.67	44.49 Bb	3.00	20.13 Bab	1.04	8.21 Aab	1.42	8.56 Aa	0.61	1.25 Aa	0.12
MT	145.31 Bab	8.54	65.43 Ba	10.12	56.79 Aa	8.61	51.10 Ab	5.50	19.74 Bab	1.10	6.99 Aab	1.90	9.41 Ba	0.50	1.14 Ba	0.06
HT	156.59 Ba	13.16	43.12 Ba	12.41	38.28 Ba	3.09	61.28 Bb	5.40	18.53 Bb	1.29	4.40 Bb	0.86	8.73 Ba	0.31	1.00 Aa	0.08
I	154.75 Ba	12.64	37.41 Ba	8.80	56.06 Aa	10.05	94.83 Aa	15.90	26.71 Ba	4.77	10.72 Ba	1.40	6.01 Bb	0.48	1.44 Aa	0.22

The capital letter indicates differences between elements in unwashed and washed material (*t*-Student test, *p* = 0.05, *N* = 8), and the lower case indicates differences between the sampling points (LSD test, *p* = 0.05, *N* = 8). (LT, low traffic; MT, medium traffic; HT, high traffic; I, post-industrial)

Iron was found in the highest concentrations in washed and unwashed linden leaves. It is a crucial metal in cell energy transformations, and its deficiency affects many physiological processes in plants. Its uptake is metabolically controlled and depends highly on soil pH, Ca, and P concentration and several heavy metals’ ratios. Toxicity symptoms occur at concentrations over 3080 mg kg^−1^ (Kabata-Pendias and Pendias 2001). In Katowice, Fe concentrations in washed linden leaves ranged from 101.82 to 215.58 mg kg^−1^ and in unwashed from 220.50 to 1291.16 mg kg^−1^. In the case of this element, an increasing trend can be observed in the mean concentrations determined in unwashed leaves in relation to traffic intensity and in the post-industrial area. The mean concentrations of Fe determined in the LT and MT areas were statistically significantly lower than in the high-traffic and industrially-related areas. This relation is also seen in other cities and plant species (Alexandrino et al. 2020; Turkyilmaz et al. 2020; Molnár et al. 2020). However, the concentrations differ in a large spectrum, depending on tree species. Aničić Urošević et al. (2019) found concentrations of Fe 101–238 mg kg^−1^ in unwashed *T. cordata* leaves in urban parks of Belgrade, and the content differed between years of study. The Fe originates mainly from geogenic sources such as soil dust particles and runoff water but also from anthropogenic activities such as municipal construction works as well as abrasion of tire and wear and brake lining tear and is commonly associated with vehicular pollution (Bisht et al. 2022).

The highest Al concentrations in unwashed and washed small-leaved linden leaves were 584.67 and 122.67 mg kg^−1^, respectively; the lowest were 10.95 and 5.96 mg kg^−1^. The mean values of the concentrations of this element in the unwashed and washed material show no statistically significant differences. In Alexandrino’s et al. (2020) study of unwashed *Araucaria heterophylla* needles from Quito, Mexico, higher levels of Al were connected to higher traffic intensity. In Belgrade, Serbia, the unwashed *T. cordata* leaves accumulated 323–235 mg kg^−1^; in washed leaves, the concentrations varied between 100 and 161 mg kg^−1^. Also, there were no significant differences between the heavy traffic areas and control sites (Tomašević et al. 2011). Similarly, no differences were found in the Al content of *Acer pseudoplatanus* leaves from Vienna at different land classes (Simon et al. 2011). The solubility of Al in soil minerals is enhanced in an acidic environment (Rengel 2004), while the soil pH at the sampling sites was close to neutral (data not shown); thus, the mobility of Al was low. This situation is typical for urban soils due to the widespread deposition of construction waste based on lime to the soil (Greinert 2015) and points out that the Al in the urban environment originates mainly from vehicular traffic dust or industrial activity.

The next highest concentration in the collected material is Mn. This element was reported in relatively high concentrations in Katowice road dust compared to other cities worldwide (Rybak et al. 2020). Mn is essential in plants’ oxidation–reduction process and N assimilation (Kabata-Pendias and Pendias 2001). Deficiency in Mn is considered below 10–30 mg kg^−1^, and the toxic concentrations highly depend on plant species (Alejandro et al. 2020). The maximum determined concentration in the unwashed material was 198.93, and the lowest was 25.72 mg kg^−1^; in the washed material, 129.95 and 16.82 mg kg^−1^, respectively, and the lowest concentrations considered in both cases were the HT points. The availability of Mn for plants depends strongly on the pH and redox conditions of the soil. With lower soil pH, the bioavailable fraction of Mn increases. Mn is present in soil in many forms (Mn (II), Mn (III), and Mn(IV), but only the reduced form is stable in solution therefore reducing soil conditions such as waterlogging or soil compaction promotes the availability of Mn (Reichman 2002). Therefore, additional data should be considered when analyzing its content in plant tissues. For example, in Bor (Serbia), the Mn content in washed linden leaves differed greatly depending on the sampling point, with the highest values in town parks (724 mg kg^−1^) and lowest in rural areas (Serbula et al. 2013). On the contrary, in Debrecen, Hungary, the value of Mn in rural areas in *T. europaea* leaves was the highest (99.7 mg kg^−1^) and at urban sites the lowest (31.4 mg kg^−1^) (Molnár et al. 2020). In *Araucaria heterophylla* needles, the Mn content also varied considerably from 1250.95 mg kg^−1^ in high-traffic sites to 224.49 mg kg^−1^ at moderate-traffic sites (Alexandrino et al. 2020); this trend was also reported in Vienna in *A. pseudoplatanus* (Simon et al. 2011).

Zn is an essential element with a natural concentration of 10–150 mg kg^−1^ (Padmavathiamma and Li 2007). Chwil et al. (2015) reported the Zn content in *T. cordata* leaves at unpolluted stands between 13.9 and 25.3 mg kg^−1^. In this study, the concentrations analyzed in washed leaves varied between 31.6 and 189.6 mg kg^−1^, with a mean value of 62.9 mg kg^−1^. However, phytotoxic concentration was found only at one sampling point. In unwashed leaves, the mean value of Zn in tissue was 90.9 mg kg^−1^, ranging from 43.9 to 260.2 mg kg^−1^. The highest Zn concentrations were found at I and HT sites in both the washed and unwashed material. The lowest average Zn concentrations were determined in LT areas and were statistically significantly different from I areas. For washed material, statistically significant differences were found in HT areas. Zn is often associated with high traffic (Alexandrino et al. 2020; Santos et al. 2019; Turkyilmaz et al. 2018). Its concentrations varied considerably depending on the species and city where samples were collected, e.g. in *Araucaria heterophylla* needles, an average of 60.43 mg kg^−1^ (Alexandrino et al. 2020), *T*. *tormentosa* leaves in Ankara 7.32 mg kg^−1^ d.m. (Turkyilmaz et al. 2018), *T. cordata* leaves in Lublin, Poland 28.1–30.8 mg kg^−1^ (Chwil et al. 2015), *Acer platanoides* (32.3 mg kg^−1^), *Fraxinus excelsior* (22.7–30.6 mg kg^−1^), *T. tormentosa* (19.3–20.85 mg kg^−1^) in Budapest, Hungary (Hrotkó et al. 2021). These results show that the concentration of Zn in the environment of Katowice city can be assessed as generally elevated since the values noted even at the sampling points at the residential areas with small traffic intensity show higher values than those indicated for other bigger European cities. Also, Rybak et al. (2020) found considerably high concentrations of Zn (2683 mg kg^−1^) in the Katowice road dust, several times higher than in other mentioned cities worldwide. Besides traffic emissions connected to tire and brake wear, in the iron industry, Zn is emitted during the galvanization process to protect steel against rusting (Giordano et al. 2024). In Katowice, since the beginning of the nineteenth century, several iron and zinc smelters existed, the number of which fell dramatically in the 1980s and 1990s, and the elevated level of Zn in the urban environment probably is still the effect of the past activities.

Similarly, the concentration of Pb in linden leaves in Katowice exceeds values typically reported for city centres. The typical concentration of Pb in plants varies between 0.5 and 10 mg kg^−1^; the values 30–300 mg kg^−1^ are considered phytotoxic (Padmavathiamma and Li 2007). In Katowice, the values in washed leaves ranged between 11.6 and 51.7 mg kg^−1^; in unwashed leaves, they ranged from 19.33 to 133.6 mg kg^−1^. The mean values of lead concentrations were highest in the post-industrial district Katowice-Szopienice in both washed and unwashed material. Generally, the concentration of Pb in unwashed material was significantly higher than in the washed material. The values obtained for the linden were higher than for the *Acer pseudoplatanus* and *A. platanoides*, also from Katowice (Steindor et al. 2016). Similar results as for the linden from Katowice were recorded by Turkyilmaz et al. (2020) in unwashed leaves of *Aesculus hippocastanum* in Ankara at the dense traffic (21.28 mg kg^−1^). However, in many cases, the level of Pb in washed leaves in Katowice exceeded multiple times values reported in other European city centres, where the Pb concentrations were 0.27–0.37 mg kg^−1^, Lublin, Poland (Chwil et al. 2015); 1.36–5.00 mg kg^−1^ Plovdiv, Bulgaria (Petrova et al. 2017); or 0.8–2.11 mg kg^−1^, Budapest, Hungary (Hrotkó et al. 2021).

Chromium content in unwashed linden leaves was determined in the range from 19.85 to 3.69 mg kg^−1^ d.m. in washed material; it ranged from 17.84 to 1.36 mg kg^−1^. In washed leaf tissue, the highest mean contents were determined in material from the I area and the lowest in HT areas; however, the differences were not statistically significant. The normal concentration of Cr in plant leaves is 0.1–0.5 mg kg^−1^, and excessive or toxic concentrations are between 5 and 30 mg Cr kg^−1^ (Kabata-Pendias and Pendias 2001). Values above 5 mg kg^−1^ were found in most of the samples from the I site (7 out of 8, in 6 over 10 mg kg^−1^). The values in unwashed leaves at most of the Katowice sampling sites exceeded the values found in unwashed leaves of *T. cordata* in busy streets of Bilbao, Spain (Soba et al. 2021), or urban parks of Belgrade (Aničić Urošević et al. 2019). Also, lower contents of Cr in unwashed leaves of different tree species from Hefei, China, were reported (*Cinnamomum camphora*, *Platanus orientalis*, *Osmanthus* sp., and *Photinia x fraseri*) (Fang et al. 2021). Only in the case of HT and I were differences between unwashed and washed leaves significant, suggesting that Cr dominates in dust at these types of locations. The presence of Cr in the environment is mainly due to anthropogenic activities such as metallurgical industries (Świetlik et al. 2011; de Conceicao Gomes et al. 2017) as well as vehicular traffic (Mihankhah et al. 2020) also in terms of pavement as a primary source of Cr in road dust (Fiala and Hwang 2021).

Cu concentrations in the washed linden leaves in Katowice varied between 3.39 and 11.24 mg kg^−1^, with the significantly lowest values at the industrial sites, and in unwashed leaves from 6.9 to 16.22 mg kg^−1^ without significant differences between other land classes. These values are considerably lower than the content found in Belgrade unwashed and washed linden leaves (64 and 21 mg kg^−1^, respectively) (Tomašević et al. 2011). Cu is an essential element for plants; the sufficient or normal concentration of Cu in plants is 5–30 mg kg^−1^ (Kabata-Pendias and Pendias 2001). The concentration in the unwashed leaves was also in line with the data obtained in similar populated Vitoria city centres in Spain (Soba et al. 2021) or Lublin, Poland (Chwil et al. 2015). Thus, the analyzed value is within the normal range. However, the concentrations of Cu in plants from the I site were at the lower demand limit for this element in plants (from 3.39 to 8.17, most of the concentrations were approximately 5–6 mg kg^−1^). Cu uptake is regulated by several factors such as soil type, particle size, organic matter, soil moisture and cation exchange capacity, and pH and relations between them. Generally, the Cu bioavailability in alkaline soils is low (Kumar et al. 2021), and the pH at the sampling points was close to neutral (data not shown).

The element with the lowest concentration in the tested tissues was cadmium. The maximum Cd concentration in the unwashed material was 11.97 mg kg^−1^. Such high concentrations were found only at one point near a busy road with a heavily used bus stop, while at the other points, the concentrations ranged from 0.87 to 2.27 mg kg^−1^. Pal et al. (2018) found the highest concentrations of Cd in road dust near the bus stations, similar to our findings, suggesting that the exhaust emissions from busses may significantly enhance the Cd content in the nearby environment. In the washed material, concentrations ranged from 0.40 to 2.27 mg kg^−1^. The mean Cd concentrations for the individual categories in washed and unwashed leaves were not statistically significantly different. Values of Cd in plant tissues considered normal are 0.05–2 mg kg^−1^, and phytotoxicity applies to values above 5 mg kg^−1^ (Padmavathiamma and Li 2007). However, the concentrations of Cd in tissues of washed leaves exceeded greatly values obtained for unwashed leaves of different tree species in Ankara (Turkey) at the dense traffic sampling points, where the maximal values reached 0.193 mg kg^−1^ (Sevik et al. 2019, Turkyilmaz et al. 2020) or industrial and urban sites (0.08 mg kg^−1^) in washed leaves of *T. europaea* from Debrecen, Hungary (Molnár et al. 2020), 0.15 mg kg^−1^ in Hefei (Fang et al. 2021), China as well as many other cities mentioned in work of Fang et al. (2021).

### Air dust element concentrations

**Fig. 4:**
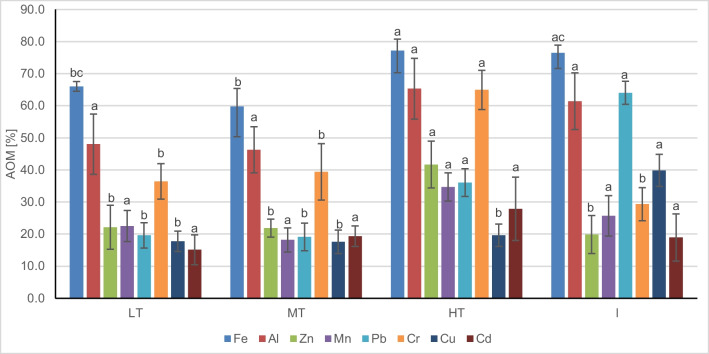
Mean values ± SE of air-originating matter (AOM) for different elements in Katowice (LT, light traffic; MT, medium traffic; HT, high traffic; I, industrial). Different letters indicate differences between each element (LSD test, *p* < 0.05, *N* = 8)

The difference between metal concentrations in unwashed and washed leaves reflects, to a large extent, the concentration of metals from dust deposited on the leaf blade (Alatou and Sahli 2019; De Nicola et al. 2008; Deljanin et al. 2016) and the AOM index demonstrates the disparity between the values of uptake of heavy metals by plant tissues and the airborne particles deposited on the leaves surface (Safari et al. 2018, Hatami-Manesh et al. 2021). The mean values of the AOM index at the different land categories are presented in Fig. 4. We also used the AOM index to visualize the possible spatial air pollution level in Katowice (Fig. S1-S3).

The highest proportion of heavy metal derived from the air in Katowice was Fe, with a minimum value of 29% and a maximum of 86% on city centre roads (Fig S1). The highest proportion of Fe in dust concerned HT and I sites. The spatial distribution of Fe AOM confirms that the Fe in the air dust in the Katowice centre originates from traffic—hot spots can be found at sampling points near busy roads and tramway lines, railways, and general post-industrial areas.

The next element highly represented in dust particles on the leaves of *T. cordata* was Al. The mean value of the AOM index in Katowice was 55%, varying from 20 to 98%, and again, analyzing the spatial distribution map, the points with high traffic seem to be a source of this metal. The areas of low traffic, such as residential areas in the South and South-East parts of the city centre, show a lower percentage of Al in the dust removed from the leaves; however, the mean values calculated for the distinguished groups of sampling points did not differ statistically significantly (Fig. S1., Fig. 4.). The generally high percentage of Fe and Al in the dust is probably also connected to the fact that they are one of the most abundant trace elements in the lithosphere (Kabata-Pendias and Pendias 2001). Also, a significant percentage of Cr collected by the leaves originated from the air. The mean value of AOM for Cr was 44%; the lowest values ranged from 7.4 to 17% in the central investigated area, with lower traffic, while 60–80% was found mainly in material from busy streets. The mean Cr AOM value at the HT was significantly higher than at the rest of the sites (Fig. S1, Fig. 4).

Among elements with relatively high AOM was Pb, with an average value of 34%. The highest proportion of Pb originating from air pollution was almost exclusively considered the post-industrial site, which ranged at 61–73%. In the centre of Katowice, points with high Pb AOM (but reaching 40–50%) are near roads with heavy traffic. The mean values of Pb at the I and HT sites are also significantly higher than at MT and LT locations (Fig. S2, Fig. 4).

The mean value of AOM for Zn, Mn, Cu, and Cd ranges from 25 to 20%, respectively. The spatial distribution, however, differs among the elements—the highest Zn AOM index of over 40% characterized HT points, and the differences in the mean Zn AOM are statistically significant (Fig. S2). Relatively high Mn AOM can be found at sampling points with heavy traffic and few points at post-industrial sites (Fig. S2), but the differences in the mean values are not significant (Fig. 4). Cu originating from air dominated at the I sites, significantly higher than in the other land categories. Relatively high Cu AOM was also found at a few points, with heavy traffic and railway and tramway lines nearby. Cd originating from the air was higher at the HT sites. The spatial distribution map also shows that the centre of the investigated area seems to be more affected by Cd pollution (Fig. S3), but there are no differences between distinguished groups of sampling sites (Fig. 4).

The studies of Deljanin et al. (2016) found that among investigated tree species *A. hippocastanum*, *A. pseudoplatanus*, *Betula pendula*, and *T. cordata*, the latter was the most effective in the accumulation of trace elements in leave tissue. However, in the survey of Deljanin et al. (2016), a considerably lower percentage of elements were removed by water from the *T. cordata* leaves. The chloroform treatment to remove the epicuticular wax fraction had a more significant effect on the percentage of removed elements approaching the values obtained in our survey. Our results demonstrate that *T. cordata* has a much higher potential to mitigate urban dust pollution by accumulating the trace metals in the leaf tissue and on the leaf surface.

In a study of heavy metal concentrations in road dust in the Chinese city of Panzhihua, elevated concentrations of Cd, Pb, and Zn were attributed to traffic; high concentrations of Cu and Pb occurred in areas influenced by metallurgical activities (Long et al. 2021), similarly to our research. Esfandiari and Hakimzadeh (2022) include Pb, Zn, Cr, and Cu as metals emitted by traffic and industrial activities. In general, elements such as Fe, Al, Zn, as well as Cu, Cr, or Pb show a strong association with traffic intensity, which is supported by the results of many studies on urban dust composition (Fujiwara et al. 2011; Alatou and Sahli 2019; Santos et al. 2019; Alexandrino et al. 2020).

### Functional traits

#### Photosynthetic pigment content

**Fig. 5:**
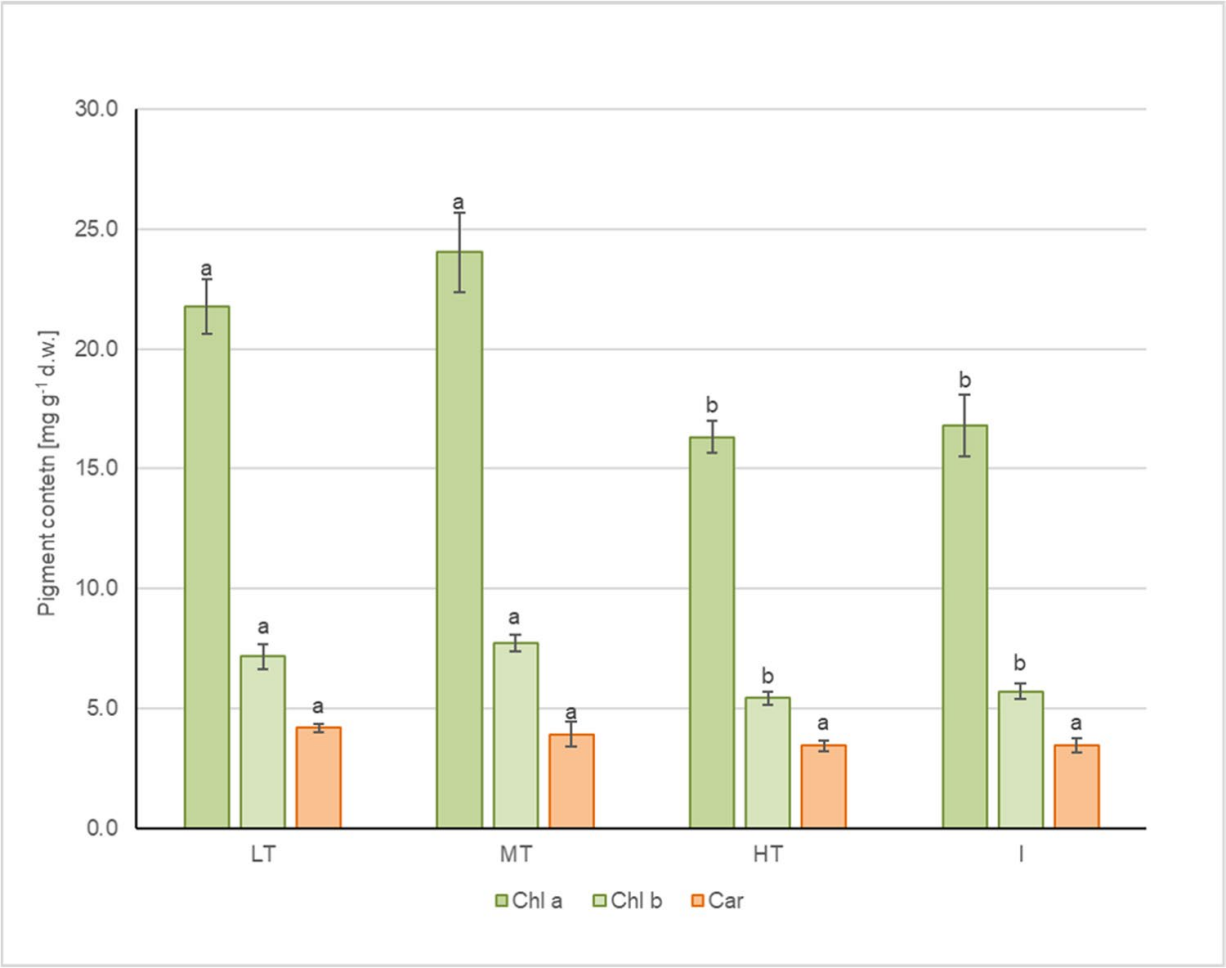
The mean ± SE value of photosynthetic pigment content in *T. cordata* from different sampling groups (LT, low traffic; MT, medium traffic; HT, high traffic; I, post-industrial). Different letters indicate differences between variants in each pigment content (LSD test, *p* = 0.05, *N* = 8)

A photosynthetic pigment, chlorophyll, is a productivity index of green plants—the reductions in chlorophyll content are linked directly to a decrease in plant growth. Also, it is most likely to be damaged by air pollution. Therefore, chlorophyll measurement is considered an important tool in biomonitoring (Rai 2016). In the case of *T. cordata* in Katowice, the mean total chlorophyll content, as well as Chl_a_ and Chl_b_, were on average lower by 27, 27, and 28%, respectively, at the HT and I sites compared to LT and MT, where the contents were the highest. The differences were statistically significant (Fig. 5). The lowest values are found near the points with heavy traffic and higher Pb, Al, and Cr levels on the spatial map of chlorophyll’s content (Fig. S4). At the same time, in the central part of Katowice’s downtown area, linden trees are characterized by higher photosynthetic pigments and lower airborne pollution of Cr, Pb, Mn, or Fe. The dust load accumulated on the leaf has been found to cause a decrease in photosynthetic pigments in several tree species in Dehli (Patel et al. 2023). Reduction in chlorophyll content is often observed due to interference of dust particles with enzymes essential to chlorophyll biosynthesis, chloroplast damage, or chlorophyll degradation (Mondal et al. 2011; Qadir et al. 2016). It can also be due to the shading effect of the dust deposited on a leaf (Prusty et al. 2005). Reduction in plant pigments in tree leaves at high-traffic sites was also found by Khosropour et al. (2019), Uka et al. (2021), and Singh et al. (2023). Several studies, however, have shown an increase in chlorophyll content while oxidative stress induced by exposure to heavy metals (Pavlović et al. 2017, Petrova et al. 2017).

Carotenoids are photosynthetic pigments which play a vital role in protecting chlorophyll from photooxidative reactions. Similarly to chlorophyll, the mean carotenoid content was 12% reduced at the HT and I sites compared to LT, but the differences were not statistically significant. Carotenoid content, however, decreases slower than chlorophyll’s content under environmental stress (Talebzadeh and Valeo 2022). The reduction in carotenoid content was also observed under the influence of coal combustion fly ash (Qadir et al. 2016) or road traffic (Prusty et al. 2005; Uka et al. 2021; Singh et al. 2023).

Vehicle emissions are a combination of pollutants that contain not only heavy metals but also PM, SO_2_, or NO_x_, and VOCs (Pant and Harrison 2013), which also concern Katowice. These additional pollutants are known to decrease pigment content (Joshi and Swami 2007) and probably also affect *T. cordata* in our study.

#### Leaf functional traits

**Fig. 6:**
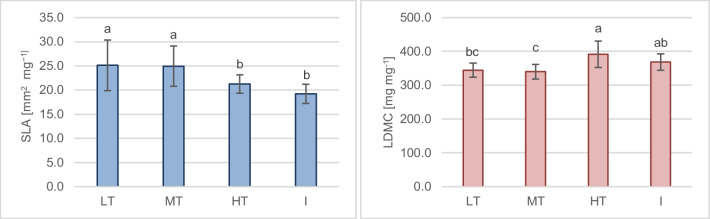
SLA and LDMC of the *Tilia cordata* leaves from the different (LT, low traffic; MT, medium traffic; HT, high traffic; I, post-industrial). Different letters indicate differences between sampling groups (LSD test, *p* = 0.05, *N* = 8)

Environmental factors affecting plants, also in the urban environment, trigger responses in the form of different adaptation strategies through changes in various traits to avoid or compensate for potential damage. Leaf density and thickness can be examples of these traits - low density of leaf tissue, and thus a larger volume of intercellular spaces and higher leaf conductivity enhances photosynthesis rates. In contrast, high leaf density suggests tougher leaves, with a higher proportion of lignified tissue or a large fraction of mesophyll cells promoting plant survival (Poorter et al. 2009). An increase in leaf density and thickness by investment in mechanical defence tissues is also considered a mechanism that occurs in stressful conditions (Wen et al. 2004; Wuytack et al. 2011). Victório et al. (2020) showed that SLA is an important indicator of the impact of heavy metals on plants, which increased with the presence of pollutants. El-Khatib et al. (2020) also found a similar effect caused by heavy metal accumulation on the SLA of two tree species, *Ficus nitida* and *Eucalyptus globulus*; SLA was higher in the areas affected by industry and traffic; the pollution was also contributing to a decrease in leaf area. In our study, however, *T. cordata* leaves show a statistically significant decrease in SLA with the traffic intensification and at post-industrial sites, while the LDMC shows the opposite trend (Fig. 6). Our findings are consistent with other research. Maisto et al. (2013) reported a decrease in SLA at the motorway in *Quercus ilex*; however, LDMC was not affected. SLA was found to be negatively correlated, and LDMC positively correlated with the particulate matter in the survey on *Euonymus japonicas* (Zhu and Xu 2021). A decrease in leaf area in urban conditions was also observed in *Platanus orientalis* (Pourkhabbaz et al. 2010; Khosropour et al. 2019) and was attributed to a strategy to minimize the absorption of pollutants.

#### Plant resources strategy

The N% and C% content in *T. cordata* leaves is presented in Table 2. The lowest N content characterized the I and HT trees, while the C content did not differ statistically significantly. Therefore, the C:N ratio was the highest for the I site trees. Considering these factors, *T. cordata* plants with higher SLA and lower LDMC, in this case from LT and MT sites, represent a resource acquisition strategy (with higher LA allowing the capture of more light and higher N content). In contrast, the resources conservation strategy dominates at the HT and I sites (leaves have lower LA and thicker leaf blades). The highest C:N ratio indicates lower foliar nutrient quality at I sites, as the N% was the lowest. Increment in the C:N ratio was also observed in plants growing in the proximity of roads in California, which was linked to elevated HM concentrations (Khalid et al. 2018). A reduction in N and protein content in plants was also reported by Ghani (2010) after HM treatment. HM (Cd, Pb, Cu, Fe, Ni) are known to hamper the enzymes involved in nutrient metabolism, including nitrogen (DalCorso 2012).

**Table 2:** N and C content (mean ± SE) and their ratio in leaves of *Tilia cordata* from the different land classes. (LT, low traffic; MT, medium traffic; HT, high traffic; I, post-industrial). Different letters indicate differences between sampling groups (LSD test, *p* = 0.05, *N* = 8)

	% *N*	SE	% C	SE	C:N	SE
LT	3.11 a	0.10	45.73 a	0.20	14.76 a	0.43
MT	3.12 a	0.09	45.45 a	0.54	14.63 a	0.57
HT	2.91 ab	0.07	45.15 a	0.21	15.58 ab	0.41
I	2.83 b	0.06	45.84 a	0.30	16.21 b	0.35

#### Relative water content

**Fig. 7:**
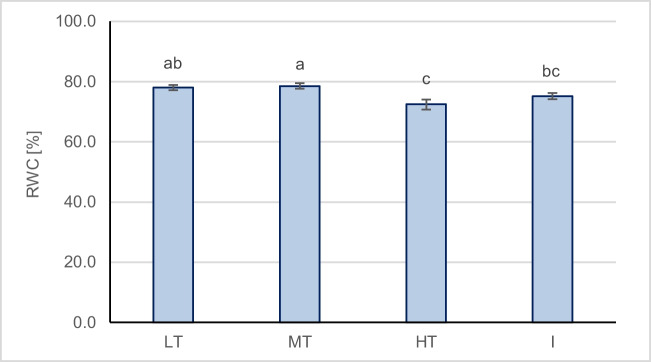
Mean ± SE values of relative water content (RWC) in leaves of *T. cordata* from different sampling groups (LT, low traffic; MT, medium traffic; HT, high traffic; I, post-industrial). Different letters indicate differences between sampling groups (LSD test, *p* = 0.05, *N* = 8)

The reduction of SLA and chlorophyll contents near the roads and heavily urbanized areas, besides pollution levels, might also result from water stress conditions, one of the most prominent urban stress factors (Lüttge and Buckeridge 2023). The land pavement was found to increase surface and air temperature and decrease humidity, as well as the photosynthesis of *Fraxinus chinensis* and *Ginkgo biloba* (Wang et al. 2019). Relative water content (RWC) is considered to be an appropriate parameter to study plant water status to drought stress (Wang 2014; Arab et al. 2023), which decreases with increasing drought (Ying et al. 2015; Boussadia et al. 2023). Pita and Pardos (2001) observed a decrease in the SLA of *Eucalyptus globulus* under water deficit due to the reduction of leaf area, which can be considered an adaptive response in high temperatures. Our results of RWC in *T. cordata* also show lower values at the HT and I sites (Fig. 7), supporting this theory, and the SLA was statistically significantly positively correlated with RWC (*r* = 0.53, *p* < 0.05). Similarly, the chlorophyll content was significantly positively correlated with RWC (Chl_a_ *r* = 0.68, Chl_b_ *r* = 0.65, Chl_tot_ *r* = 0.68, *p* < 0.05). Drought stress caused the reduction and decomposition of chlorophyll in *Frankenia* leaves (Chegah et al. 2013). Also, the reduction in chlorophyll content in *T. cordata* trees was an effect of drought stress in an experiment by Kalaji et al. (2018). The study by Zhao et al. (2019) showed that drought stress in *Peonia ostii* downregulated chlorophyll metabolism by inhibiting the activity of critical enzymes of chlorophyll biosynthesis.

Excessive concentrations of HM can also induce disturbances to water status in plants. High concentrations of Pb and Ni cause water deficit in plants by reducing the transpiration rate, altering the osmotic pressure of the cell sap and the water potential of the xylem, which negatively changes the plant water status (DalCorso 2012). Thus, the causes of the observed disturbances are complex and must be explained by a set of unfavourable factors related to the urban environment.

#### Leaf damages

**Fig. 8:**
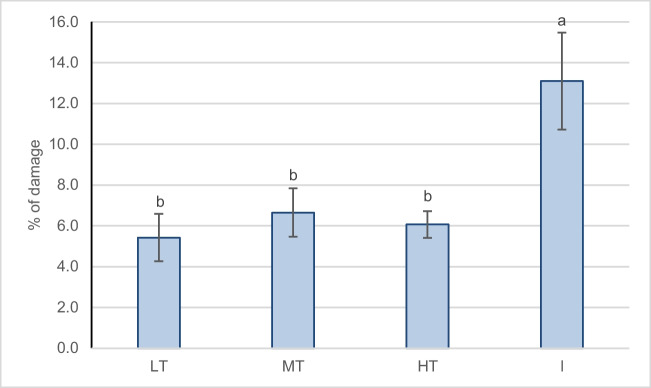
Mean percentage and SE of the damaged leaf blade of *Tilia cordata* at different sampling groups (LT, low traffic; MT, medium traffic; HT, high traffic; I, post-industrial). Different letters indicate differences between investigated groups (LSD test, *p* = 0.05, *N* = 8)

The *T. cordata* leaf damages occurred as small necrotic spots, marginal leaf necrosis, and mines left by mining larvae (see Fig. 2). The highest percentage of leaf blade damage can be observed at the I site, where it has reached an average value of 13.1% (max 25%) and differed statistically significantly between the rest of the sampling groups, where the mean value ranged from 5.4 to 6.6% (Fig. 8). The damages could be of different origins and a combination of many factors since the urban environment is characterized by several stressors such as HM, which cause alterations in chloroplast organization and plasmolysis of the leaf cells and can lead to chlorosis and necrosis visible on leaves (Sorrentino et al. 2018) or acid precipitation which causes cuticle alterations and areas of total tissue destruction (Rodríguez-Sánchez et al. 2020), as well as leaf scorching and chlorosis (Dineva 2004). Additionally, urban trees, especially street trees, are more susceptible to pests due to drought and suboptimal conditions, with little area for root expansion and disturbances (Tubby and Webber 2010).

## Summary

In this study, we have analyzed the content of chosen metals in washed and unwashed leaves of *T. cordata* affected by different levels of traffic intensity and industry. Moreover, to assess whether or to what extent urban pollution influences the functioning of the species, some functional traits were determined. The PCA and ANOVA analyses indicated that Fe, Zn, Cr, and Al are connected to traffic emissions, while Pb to industrial activities. The concentrations of elements such as Zn, Pb, and Cd are relatively high compared to other city centres worldwide, indicating that the lack of environmental policy in past decades still affects the environment, although heavy industry no longer prevails in Katowice. It is still evident, especially in Katowice-Szopienice, and can be observed on the spatial distribution maps. Besides the industrial district of Katowice, most of the elements were found in higher proportion in dust collected on leaves from the western part of the Katowice downtown at the points categorized as HT, with intensive municipal transport, road intersections, tramway lines but also with the domination of coal-fired tenements. Lower values were found in the southeastern part of the residential area with central heating and relatively low traffic. *T. cordata* has proven to have a high potential to mitigate dust pollution by trapping trace elements on the leaf surface and in the tissues; therefore, it is a beneficial tree species in urban greenery.

The concentration of chlorophylls and carotenoids was slightly reduced at the HT and I sites, which were most notably related to RWC, lower at these sites. The reduction in water content might result from the stress caused by the elevated metal content and low humidity due to a high degree of paving or probably from the combination of stressors affecting plants in an urban environment. The study also revealed a tendency to change the resources economics by the studied tree species—the individuals from less stressed parts of the city had higher leaf SLA and lower LDMC, which indicates higher photosynthesis rates and represents a resource acquisition strategy. In contrast, the trees from HT and I sites had lower SLA, higher LDMC, and a higher C:N ratio, suggesting the resource conservation strategy. These results imply that the plasticity of functional traits in response to unfavourable urban conditions exists. However, defining which environmental factor is responsible as a primary selective agent is difficult. Those changes in traits of *T. cordata* can be related to urban environmental conditions and can be used as an indicator of deterioration of the habitat quality. The knowledge of the species’ possibilities and scope of adaptation mechanisms is crucial in urban ecology assessment, urban landscaping, and greenery planning, as planting trees in cities has a significant impact on the urban environment by reducing the urban heat island and mitigating dust and gaseous pollution. There is a great need to investigate how urban atmospheric pollution affects the functional traits of plants to select the most suitable species that can overcome stressful conditions and effectively capture pollutants.

## Supplementary Information

Fig S1. Distribution patterns of air-originated metals (%) Fe, Al and Cr in *Tilia cordata* leaves in the city of Katowice. A – city centre, B – the post-industrial district of Katowice – Szopienice. (PNG 3696 kb)

High resolution image (TIFF 15850 kb)

Fig S2. Distribution patterns of air-originated metals (%) Zn, Mn and Pb in *Tilia cordata* leaves in the city of Katowice. A – city centre, B – the post-industrial district of Katowice – Szopienice. (PNG 3695 kb)

High resolution image (TIFF 16185 kb)

Fig S3. Distribution patterns of air-originated metals (%) Cd and Cu in *Tilia cordata* leaves in the city of Katowice. A – city centre, B – the post-industrial district of Katowice – Szopienice. (PNG 2475 kb)

High resolution image (TIFF 10705 kb)

Fig S4. Distribution patterns of chlorophylls (*Chl*_*a*_, *Chl*_*b*_ and *Chl*_*total*_*)* content in *Tilia cordata* leaves in the city of Katowice. A – city centre, B – the post-industrial district of Katowice – Szopienice. (PNG 3465 kb)

High resolution image (TIFF 14827 kb)

Fig S5. Distribution patterns of functional traits (SLA, LDMC) and damage percentage of the *Tilia cordata* leaves in the city of Katowice. A – city centre, B – the post-industrial district of Katowice – Szopienice. (PNG 3467 kb)

High resolution image (TIFF 14723 kb)

## Data Availability

The authors confirm that the data supporting the findings of this study are available within the article and additional information can be obtained from the corresponding author upon reasonable request.
