# Supplementary figures and images for: The effect of industrial and urban dust pollution on the ecophysiology and leaf element concentration of Tilia cordata Mill
Source: Environ Sci Pollut Res Int. 2024 Sep 24;31(48):58413–29. doi: 10.1007/s11356-024-34999-9 (PMC11467088; doi:10.1007/s11356-024-34999-9)

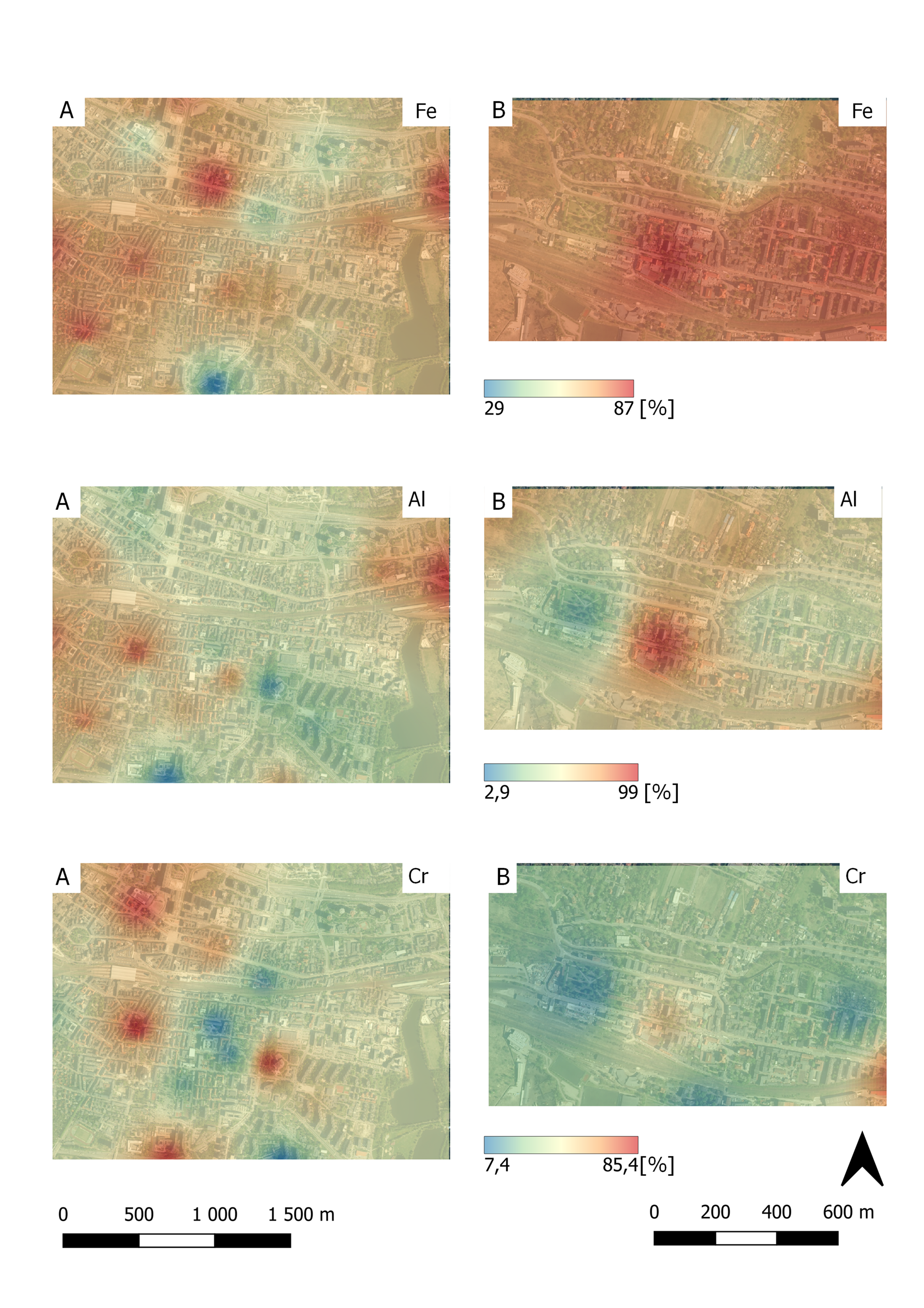

Supplement: Supplementary file 1 — Distribution patterns of air-originated metals (%) Fe, Al and Cr in Tilia cordata leaves in the city of Katowice. A – city centre, B – the post-industrial district of Katowice – Szopienice. (PNG 3696 kb) [file 11356_2024_34999_Fig9_ESM.png]

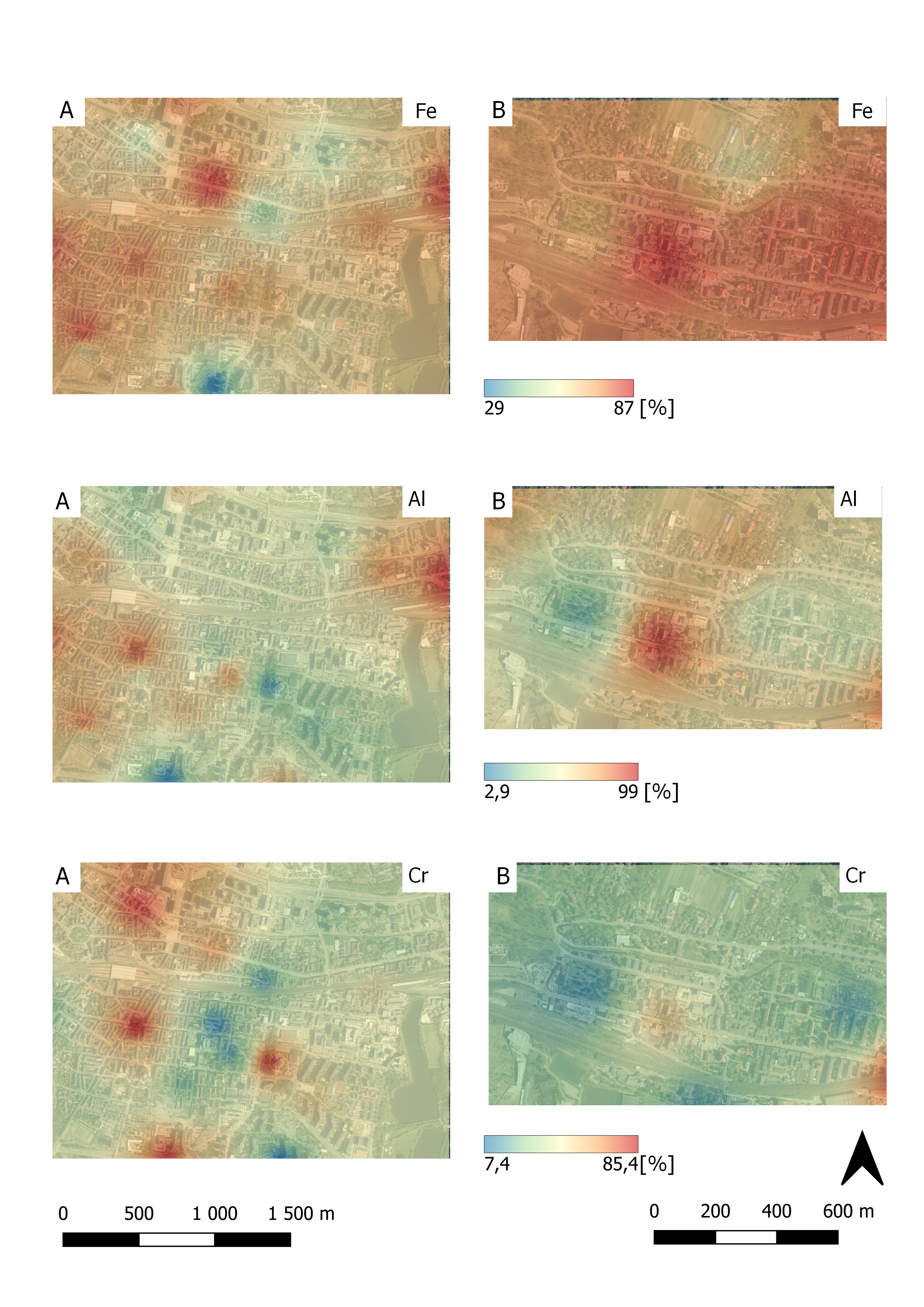

Supplement: Supplementary file 2 — High resolution image (TIFF 15850 kb) [file 11356_2024_34999_MOESM1_ESM.tiff]

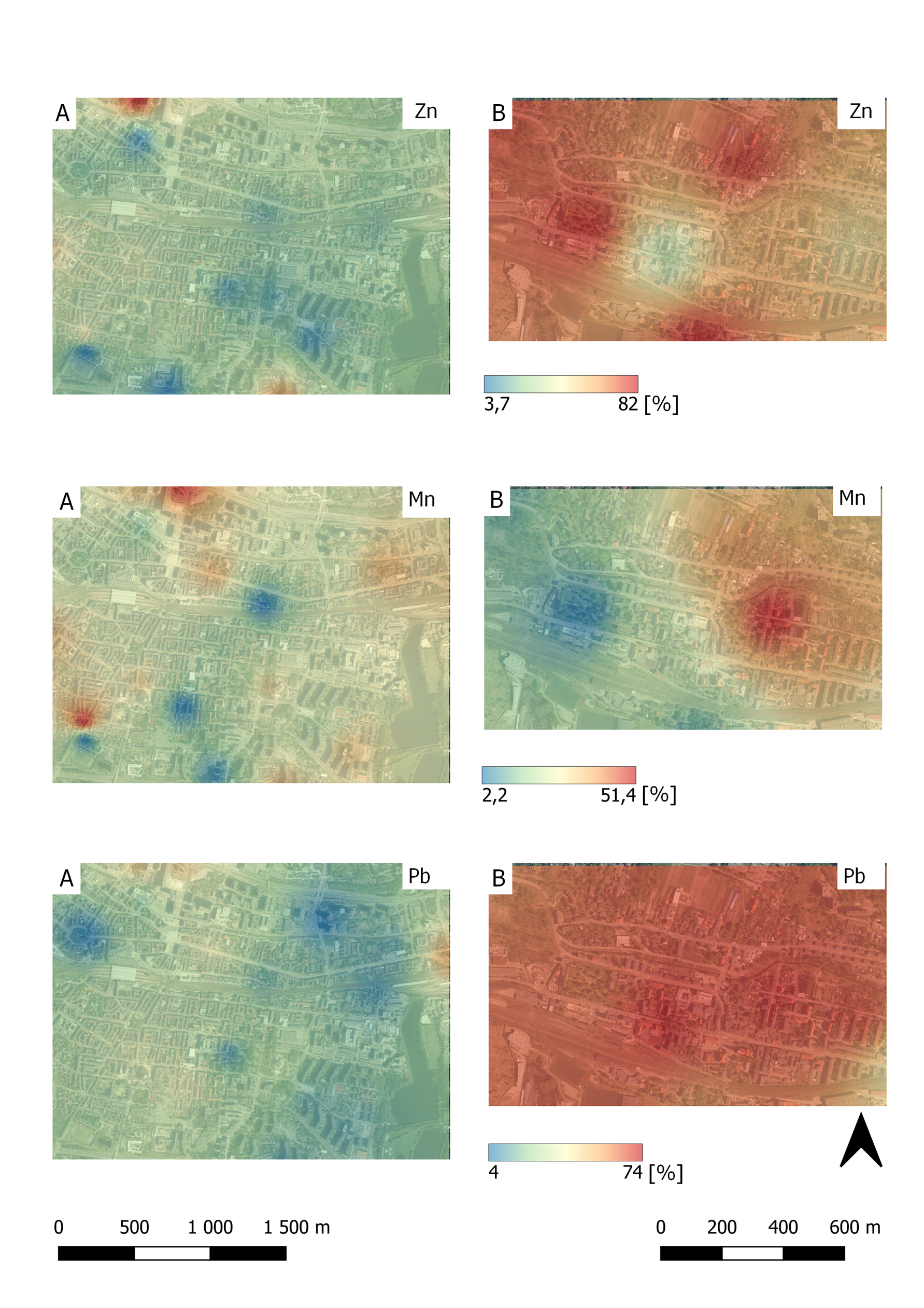

Supplement: Supplementary file 3 — Distribution patterns of air-originated metals (%) Zn, Mn and Pb in Tilia cordata leaves in the city of Katowice. A – city centre, B – the post-industrial district of Katowice – Szopienice. (PNG 3695 kb) [file 11356_2024_34999_Fig10_ESM.png]

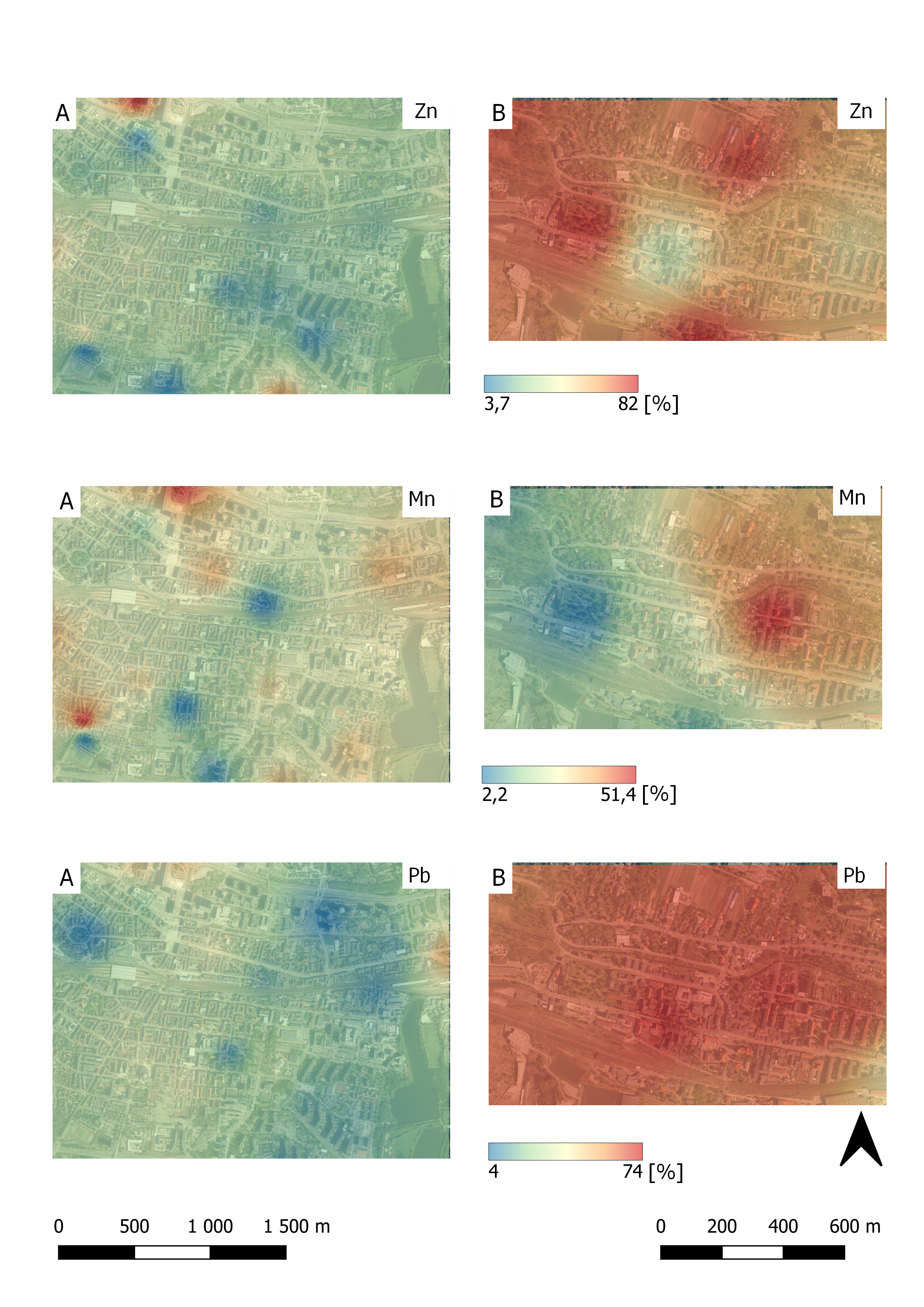

Supplement: Supplementary file 4 — High resolution image (TIFF 16185 kb) [file 11356_2024_34999_MOESM2_ESM.tiff]

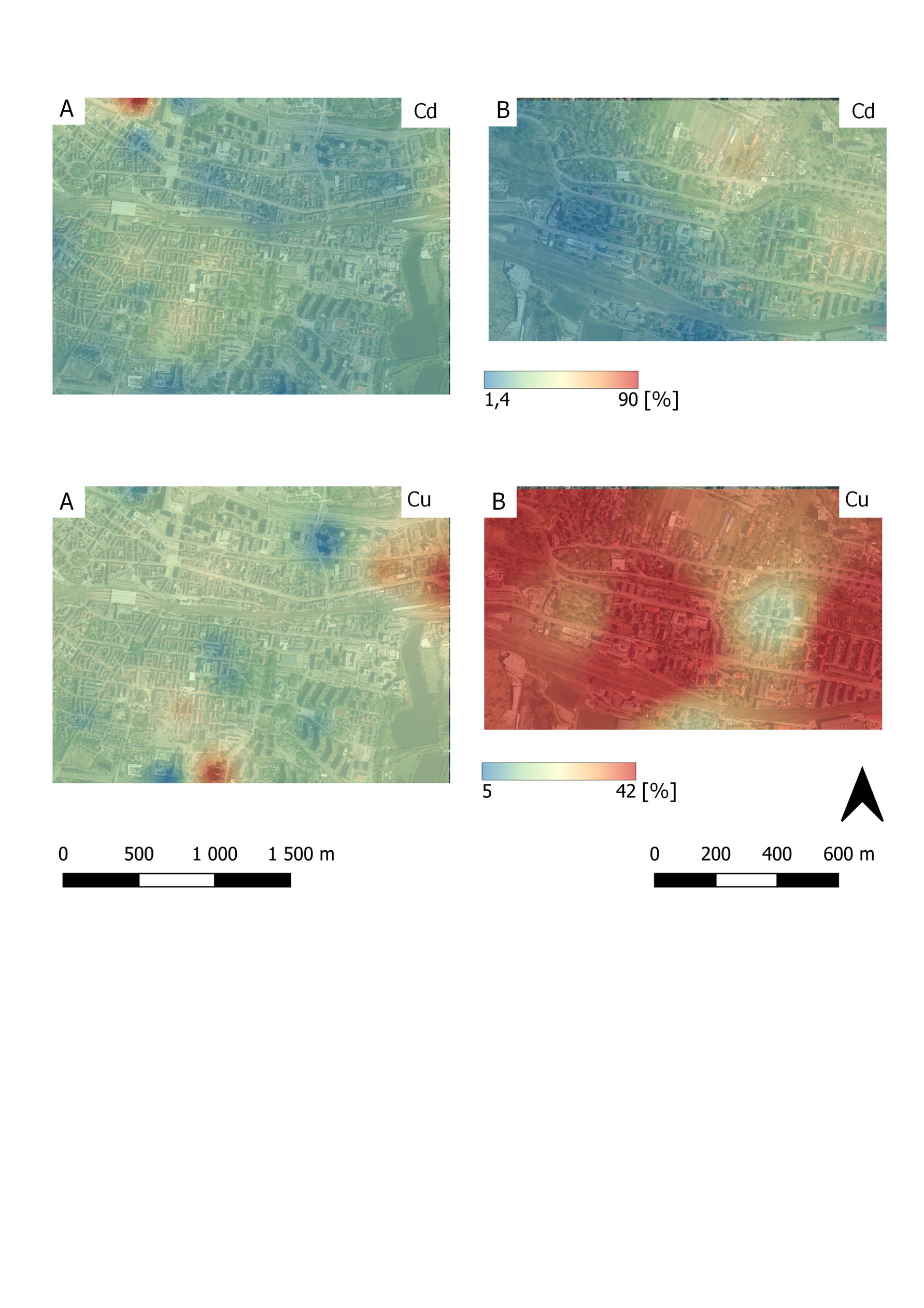

Supplement: Supplementary file 5 — Distribution patterns of air-originated metals (%) Cd and Cu in Tilia cordata leaves in the city of Katowice. A – city centre, B – the post-industrial district of Katowice – Szopienice. (PNG 2475 kb) [file 11356_2024_34999_Fig11_ESM.png]

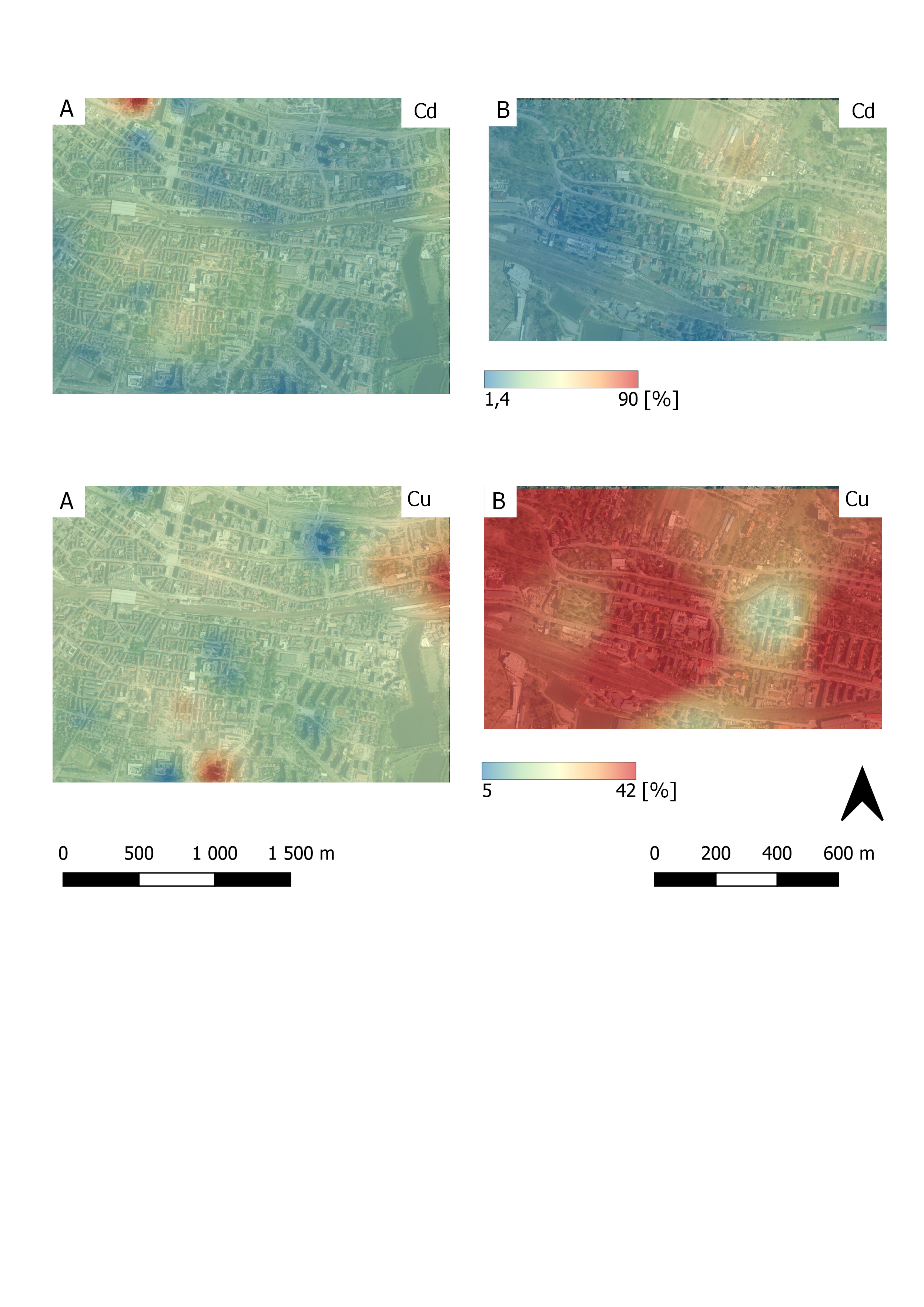

Supplement: Supplementary file 6 — High resolution image (TIFF 10705 kb) [file 11356_2024_34999_MOESM3_ESM.tiff]

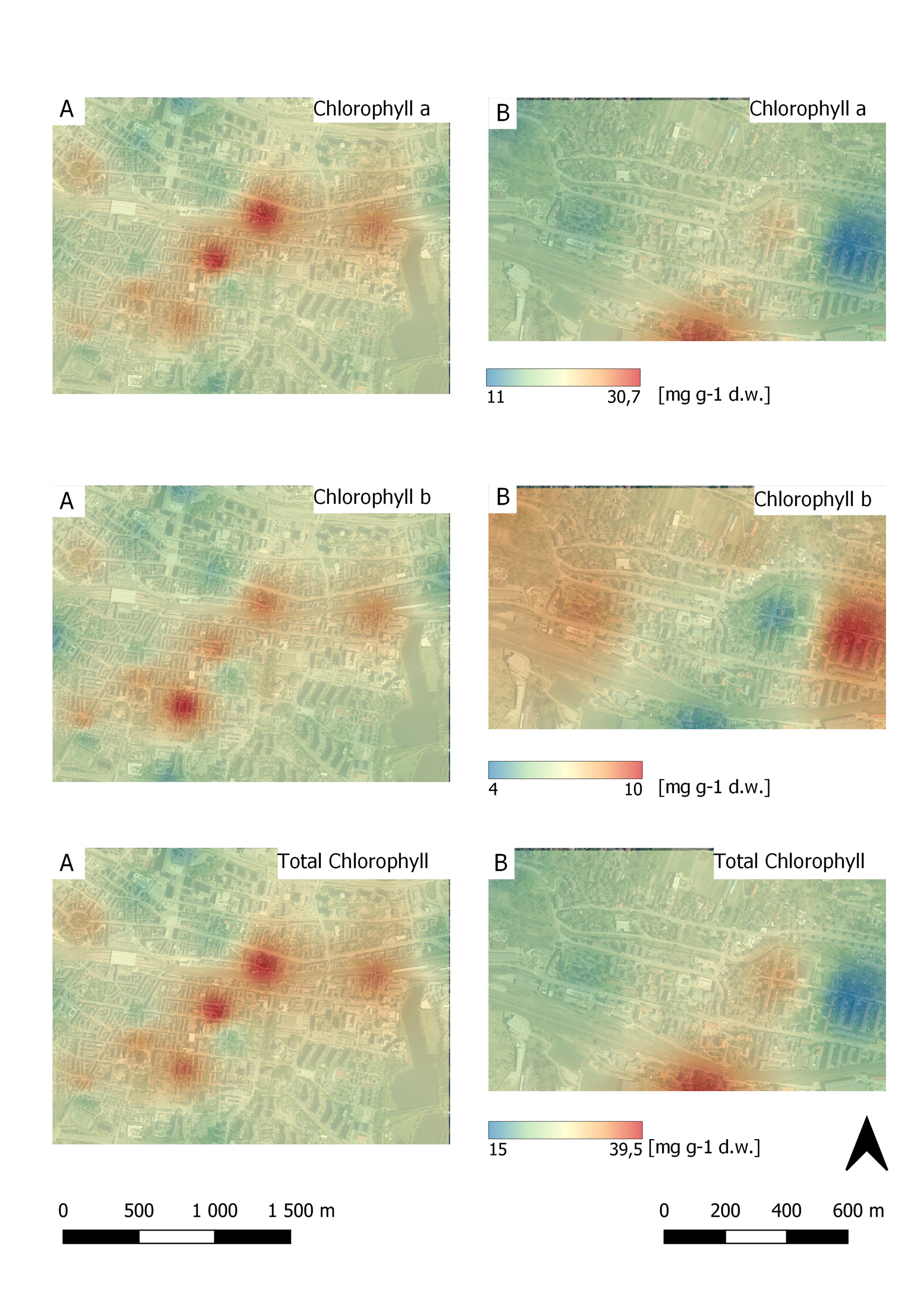

Supplement: Supplementary file 7 — Distribution patterns of chlorophylls (Chla, Chlb and Chltotal) content in Tilia cordata leaves in the city of Katowice. A – city centre, B – the post-industrial district of Katowice – Szopienice. (PNG 3465 kb) [file 11356_2024_34999_Fig12_ESM.png]

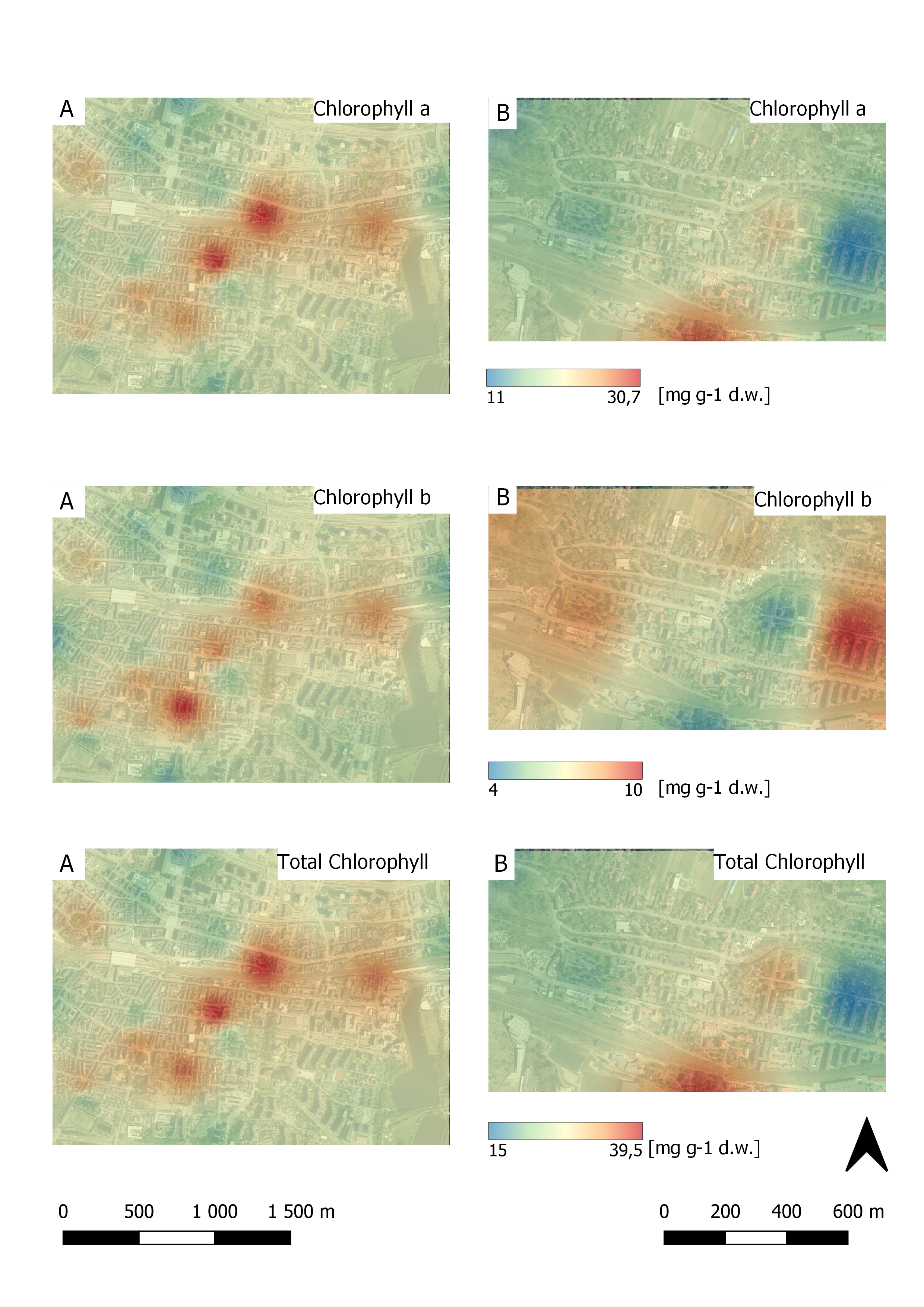

Supplement: Supplementary file 8 — High resolution image (TIFF 14827 kb) [file 11356_2024_34999_MOESM4_ESM.tiff]

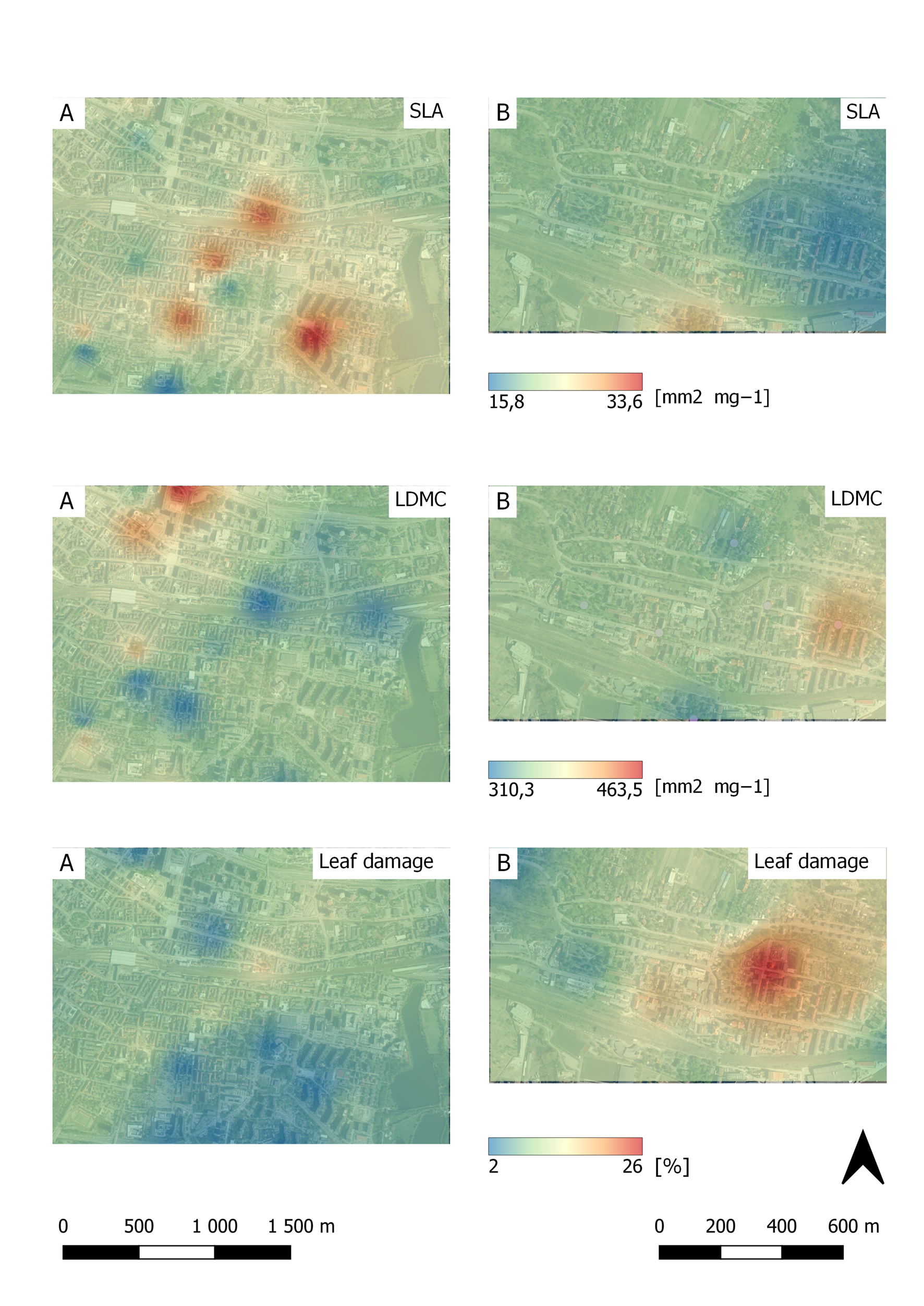

Supplement: Supplementary file 9 — Distribution patterns of functional traits (SLA, LDMC) and damage percentage of the Tilia cordata leaves in the city of Katowice. A – city centre, B – the post-industrial district of Katowice – Szopienice. (PNG 3467 kb) [file 11356_2024_34999_Fig13_ESM.png]

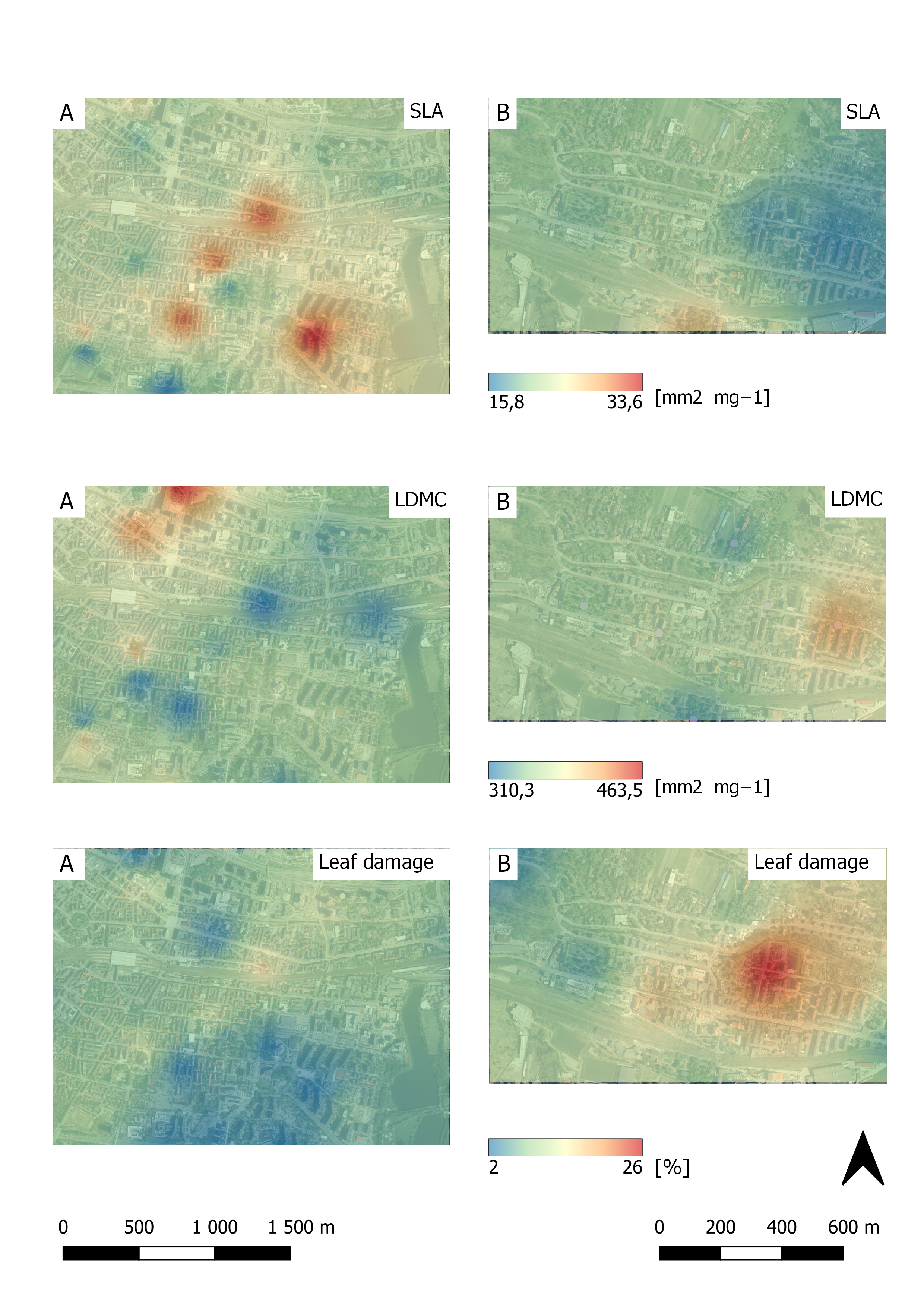

Supplement: Supplementary file 10 — High resolution image (TIFF 14723 kb) [file 11356_2024_34999_MOESM5_ESM.tiff]
